# Lung clearance index short-term variability in cystic fibrosis: a pre-post pulmonary exacerbation study

**DOI:** 10.1186/s13052-023-01574-w

**Published:** 2024-01-17

**Authors:** Matteo De Marchis, Enza Montemitro, Alessandra Boni, Alessandra Federici, Daniele Di Giovanni, Luca Cristiani, Renato Cutrera, Alessandro G. Fiocchi

**Affiliations:** 1 Pneumology and Cystic Fibrosis Unit, Bambino Gesù Children’s Hospital IRCCS, Rome, Italy; 2 University of Magna Graecia of Catanzaro, Calabria, Italy; 3 Industrial Engineering Department, University of Rome Tor Vergata, Rome, Italy; 4 Unicamillus, Saint Camillus International University of Health Sciences, Rome, Italy; 5 Allergy Division, Bambino Gesù Children’s Hospital IRCCS, Rome, Italy

**Keywords:** Cystic fibrosis, Lung clearance index, Multiple breath washout, Spirometry pulmonary exacerbation, Intravenous antibiotic therapy

## Abstract

**Background:**

Multiple Breath washout (MBW) represents an important tool to detect early a possible pulmonary exacerbation especially in Cystic Fibrosis (CF) disease. Lung clearance index (LCI) is the most commonly reported multiple breath washout (MBW) index and in the last years was used as management measure for evaluation. Our aim was to analyze clinical utility of LCI index variability in pulmonary exacerbation in CF after intravenous (IV) antibiotic therapy.

**Methods:**

A single-center study was conducted at CF Unit of Bambino Gesù Children’s Hospital among hospitalized > 3 years patients for pulmonary exacerbations and treated with antibiotic IV treatment for 14 days. MBW and spirometry were evaluated within 72 h of admission to hospital and at the end of hospitalization. Descriptive analysis was conducted and correlations between quantitative variables were investigated.

**Results:**

Fifty-seven patients (M22/F35) with an average age 18.56 (± 8.54) years were enrolled. LCI2.5 was significantly reduced at the end of antibiotic treatment in both pediatric and adult populations with an average reduction of -6,99%; 37/57 patients denoted an improvement, 20/57 are stable or worsened in LCI2.5 values and 4/57 (7.02%) had a significant deterioration (> 15%) at end of treatment. On the contrary a significative elevation of FEV1 and FVC were found, respectively of + 7,30% and of + 5,46%. A positive good correlection among LCI 2.5 and Scond (rho = + 0,615, p = 0.000) and LCI 2.5 and Sacin (rho = + 0,649, p = 0.000) and a negative strong correlation between FEV1 and LCI 2.5 were found in post treatment period. A similar modification of LCI 2.5 and FEV1 was noticed in both adult and pediatric population.

**Conclusions:**

LCI may have a role in the routine clinical care of both adult and pediatric CF patients as a good tool to assess response to IV antibiotic end-therapy in the same way as FEV1.

## Background

CF is the most common life-limiting autosomal recessive disease in Caucasian population, and is a complex multisystemic disorder caused by mutations in the gene encoding the cystic fibrosis transmembrane conductance regulator (CFTR), an ion channel which primarily regulates anion transport through cell surface. To date, more than 2000 mutations have been identified [1]. Functional failure of CFTR leads to multisystemic dysfunction, involving lungs, gastrointestinal tract, liver and pancreatic gland. Impaired mucociliary clearance and dense secretions primarily result in chronic pulmonary inflammation and infections, irreversible lung architecture modification, respiratory failure and death [2].

In the last decade, LCI started to be used in CF management as an efficacy endpoint in interventional trials thanks to its ability to effectively assess small airways disease both in preschool and middle-age children, when forced expiratory volume in 1 s (FEV1) is generally within normal range and CF lung disease still mild [3–5].

LCI is the most commonly reported MBW index in the pediatric literature and it is defined as the number of functional residual capacity lung turnovers required to reduce the alveolar concentration of a tracer-gas to a given fraction of its starting value, historically 1/40 (2.5%). A particular advantage of LCI in pediatric population is feasibility by passive cooperation and minimal coordination across all the pediatric age ranges [6].

Horsley et al. described Lung clearance index as a sensitive, repeatable and practical measure of airways disease also in adults with cystic fibrosis [7].

Despite being the gold standard for the assessment of pulmonary function in CF lung disease, FEV1 measurement through spirometry has been shown to be less sensitive than LCI in detecting early abnormalities and peripheral airways disease in CF [8]. LCI is an early marker of ventilation inhomogeneity, reflecting initial airways dysfunction in CF population with normal pulmonary function tests [9–10] and it is being recognized as a useful surrogate pulmonary outcome measure, especially in the new era of CFTR modulators, where new disease trajectories will require adequate clinical markers to characterize clinical phenotypes and monitor the efficacy of treatments [11–14].

LCI is a sensitive and feasible measures index (LCI) with strong intra-test and inter-test repeatability [15].

In CF, pulmonary exacerbations are crucial events which progressively determine a loss of respiratory function, worsening of the quality of life and negatively impact overall survival. According to studies, lung function fails to return to baseline value in up to 25% of CF pulmonary exacerbations, despite a prompt antibiotic treatment [16–17]. In contrast to studies in which FEV1 was used as primary outcome, consistently showing a positive treatment effect [18–21], studies addressing LCI as outcome measure obtained heterogeneous results [22–24]. LCI significantly increases in patients with a pulmonary exacerbation, but LCI response to therapy for pulmonary exacerbations is heterogeneous and not completely clear in literature [25].

Our primary aim was to evaluate LCI index short-term delta-variability response in pulmonary exacerbation at end of antibiotic IV therapy. We also aimed to analyze the possible correlations between spirometry and LCI values post therapy course to support treatment variability.

## Methods

### Study design

A pre-post single-center retrospective study according to STROBE statement checklist was conducted at the CF Centre of Bambino Gesù Pediatric Hospital, from September 2020 to February 2021. Fifty-eight consecutive CF patients eligible for antibiotic IV treatment with a Pulmonary exacerbation (PE) according to standard Fuchs’ criteria [26], were enrolled. We included patients starting from 3 years of age. Exclusion criteria were FEV1 ≤ 40%of predicted and chronic Burkholderia cepacia complex and Non-Tuberculous mycobateria airways infection to reduce cross infections and to reduce bias of advanced lung disease with possible reduced modifications. All study participants performed primarily multiple breath washout test and subsequently spirometric assessment within 72-hours from hospital admission for pulmonary exacerbation and at the end of IV antibiotic course. IV treatment was conducted entirely in hospital, according to the CF guidelines, providing 14 days of intravenous processing with standard chest daily physiotherapy pulmonary rehabilitation [27]. The study protocol was approved by the local ethics committee of Bambino Gesù Children’s Hospital (2819_OPBG_2022). A written informed consent was obtained from all eligible subjects or from their parents/guardians when necessary.

### Sample size

We conducted sample size calculations starting from previous study of Sonneveld N. et al. [25]. To detect a difference of 0.3, considering 0.8 SD of differences, given a type I error of 0.05% and 80% power, 58 subjects were required for the analysis. Considering that pediatric population has proportionally milder disease and with FEV1 generally in the normal range, we decided to divide the population into pediatric and pre-adult age (PPaa) with < 18 years and adult population with > 18 years age.

### Multiple-breath-washout

MBW measurements were performed with a flow, volume and molecular mass measurement analyzer (EXHALYZER D, Ecomedics, Switzerland), according to ERS/ATS Consensus Guidelines by healthcare professional. A minimum of two acceptable MBW trials for each patient at each test evaluation. Washout repeats were excluded if there was evidence of a leak or a large difference between the LCI or functional residual capacity (FRC) measurements (> 25% from the median) [28]. Normal cut-off value of LCI was set at 7.1 according 3.3.1 software (ULN for LCI) [29, 30]. As reported in literature, it was described that in the pre-school population there was a greater variation in LCI post antibiotic treatment, with an average treatment effect of − 15.5% (95% CI − 25.4 to − 5.6) [31].

Scond and Sacin, two MBW test secondary indexes of conductive airways and acinar zone respectively, collected during the measurements, were included in the analysis [32].

### Spirometry

FEV1, forced vital capacity (FVC) and forced expiratory flow 25–75% (FEF25-75) were measured using standard spirometry following the ATS/ERS guidelines [33] with an ultrasound spirometer (Ecomedics, EXHALIZER-D software 3.3.1). Data were expressed in % predicted using the normative data from the Global Lung Function Initiative software (GLI 2012, Global Lung Function Initiative Task Force).

### Statistical analysis

The collected data, related to demographic and clinical characteristics of patients, were presented as counts and proportions (categorical data) or mean, median, standard deviation (continuous data). The t-Student paired test was used to compare normally distributed continuous data and Wilcoxon signed-rank tests for data that are not normally distributed. In line with the secondary objectives of the study, correlations between quantitative variables were investigated using Pearson’s (parametric) or Spearman’s (non parametric) correlation coefficient. Considering a significance level of 0,05 Bonferroni corrected P-value has been determined on the basis of number of tests performed. The statistical elaboration of the data was performed using IBM SPSS Statistics (version 25).

## Results

Fifty-eight patients with CF (M/F 22/36) were recruited but one was left during follow-up; their mean age (± SD) was 18.56 ± 8.7 years for females and 18.57 ± 8.5 years for males. PPaa patients were 28/57 with an average age of 11,23 ± 3.95 years, and adult patients were 29/57 with an average age of 25,65 ± 5.01 years. The majority of the subjects in the sample were pancreatic insufficient and ΔF508 homozygous. Pseudomonas colonization was present in 44% of patients and 17% of sample subjects were treated by CFTR modulators. The demographic and anthropometric characteristics of the entire study sample are shown in Table 1.

**Table 1:** **Demographic and clinical characteristics of the study sample (57 patients)**

Demographic / clinical characteristic..	Value
Sex (M/F)	22/36
ΔF508 homozygous (%)	15
ΔF508 heterozygous (%)	85
Age (yrs) ± SD	18.56 (8.54)
Height (cm) ± SD	150.16 (20.63)
Weight ± SD	46.11 (16.87)
BMI ± SD	19.69 (3.49)
Chronic pseudomonas aeruginosa colonization (%)	44%
Pancreatic insufficiency (%)	96.5%
FEV1 (average) ± SD	73.48(21,20)
Patients on CFTR modulator (%)	17%

Considering respiratory function at baseline, within the sample subjects had a moderate impairment with mean FEV1 of 73.48 (± 21,19) and mean LCI 2.5 of 12.85 (± 3.86) as shown in Table 2. Modification after antibiotic IV therapy was statistically significative for FEV1 (p = 0.000), FVC% (p = 0.000) and for LCI 2.5 (p = 0.001); no differences were found for Sacin (p = 0.33) and Scond (p = 0.38). In particular, FEV1 + 7,30% and FVC % +5,46% variations instead a -6,99% reduction of LCI2.5 were found at end of antibiotic IV treatment.

Regarding LCI 2.5, 37/57 patients showed an increase in LCI after therapy with an average of -8.41% (in particular 13 patients out of 37 improved their LCI value over the cut-off considered of 15%) while in 20/57 remained stable or worsened.

In particular, the majority of this population (patients with stable or worsened LCI value) was female (13/20) with an average age of 16.94 years (± 8.04) and especially with pseudomonas colonization (11/20). Regarding genetic profile, the most of population was with ΔF508 and minimal function CFTR mutation (16/20) and with a good lung function (FEV1 > 40% of predicted) with an average FEV1 of 80.71% (± 20,64) as shown in Table 2.

**Table 2:** Characteristics of population with stable or worsened LCI value (20 patients) in comparison with population with improvement LCI values (37) post intravenous antibiotic treatment

Characteristics of population	Patients with stable or worsened LCI post IV Value (Number)	Patients with improvement LCI post IV Value (Number)
Sex (M/F)	7/13	15/22
ΔF508 homozygous (%)	3 (15%)	7 (18.9%)
ΔF508/Minimal Function	16 (80%)	20 (54.1%)
Minimal Function/ Minimal Function	1 (5%)	5 (13.5%)
Age (yrs) ± SD	16.94 (8.04)	21,18 (8.46)
FEV1 > 70%	12	21
FEV1 40–70%	8	16
FEV1 (average) ± SD	80.71(20.64)	70.63 (20.96)
Chronic pseudomonas aeruginosa colonization	11	22
Chronic Staphylococcus colonization	6	10
Others	3	5

**Fig. 1:**
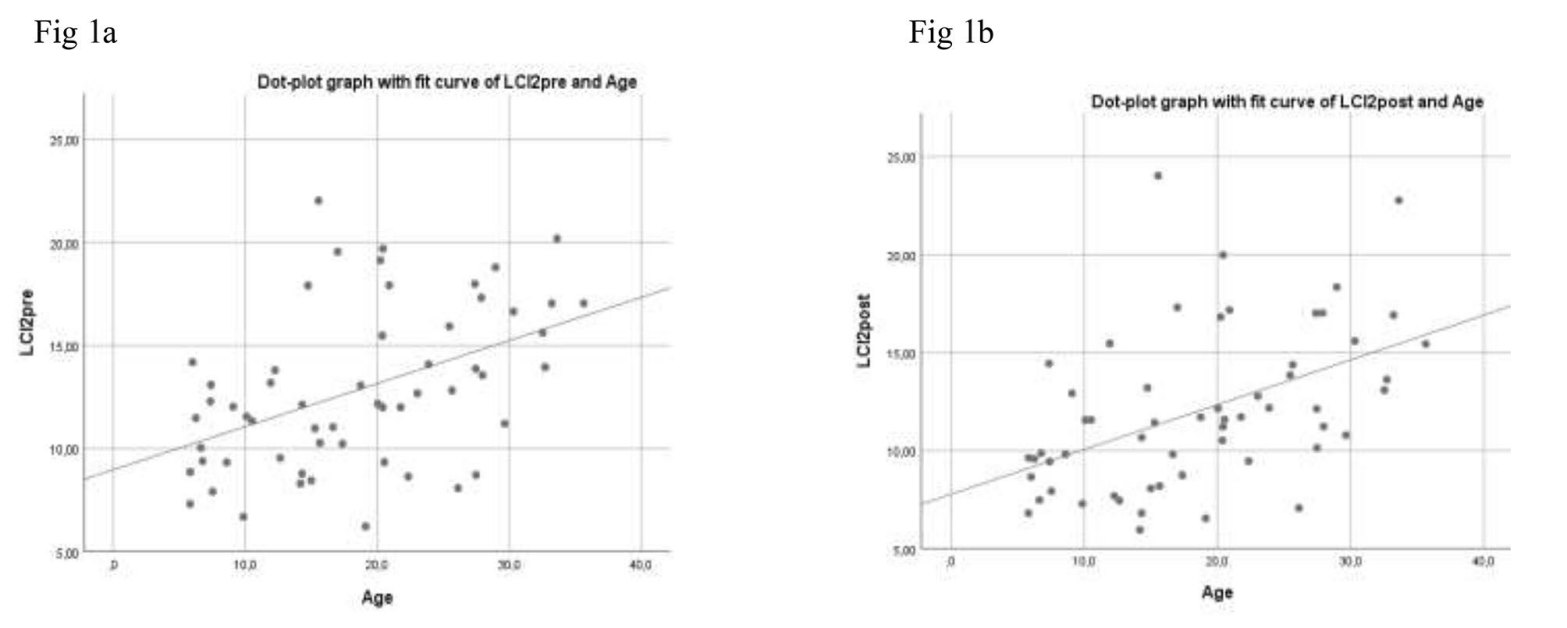
(**1a** and **1b**) show Correlation between values of LCI and age before and after the administration of intravenous antibiotic therapy. The figure shows an important information about the degree of dispersion of LCI value and it is clear that LCI values increase directly proportional to age of patients

Among total sample, 4/57 patients showed a significant deterioration > 15% in LCI 2.5 after antibiotic therapy. Figure 1a and b show a correlation between values of LCI and age before and after the administration of intravenous antibiotic therapy; it is clear that the values of LCI are higher in adult population than pediatric population.

In addition all differences in the measured pre and post antibiotic IV therapy respiratory parameters are shown in Table 3.

**Table 3:** Spirometry and multiple breath washout parameters of the study sample (mean value ± SD ); statistically significant changes in bold

Multiple Breath washout average values	Pre (mean ± SD)	Post (mean ± SD)	Variation pre-post (%)
LCI 2.5	12.85 (3.86)	12.01 (4.05)	**-6,99**
LCI 5	7.72 (1.98)	7.34 (2.03)	-5,18
Second	0.08 (0.10)	0.07 (0.04)	-14,29
Sacin	0.223 (0.16)	0.246 (0.20)	+ 9,35
**Spirometric parameters**	**Pre (mean ± SD)**	**Post (mean ± SD)**	
FEV1 (liters)	2.08 (0.85)	2.23 (0.89)	+ 6,73
FEV1	73.48 (21,19)	79.27 (20.21)	+ 7,30
FVC (liters)	2.70 (1.06)	2.83 (1.13)	+ 4,59
FVC %	81.83 (16.67)	86.56 (16.43)	+ 5,46
FEF 25–75 (Liters/second)	1.99 (1.14)	2.07 (1.12)	+ 3,86
FEF 25–75%	62.3 (33.0)	67.2 (31.5)	+ 7,29

Regarding sub-classification of the sample, we noticed a similar modification after IV antibiotic treatment for PPaa and adult subjects in FEV1 (-6,36% vs. -5,25%) and in LCI 2.5 (+ 1,06 vs. + 0,62).

Considering correlation, we noticed a positive good correlation among LCI 2.5 and Scond (rho = + 0.615, p = 0.000) and Sacin (rho = + 0.649, p = 0.000) in post treatment period, instead FEV1 and LCI 2.5 showed a negative strong correlation (rho= -0.78 with p = 0.000) after IV antibiotic therapy.

## Discussion

According to our results, LCI values appear to significantly vary during the course of a PE in people with CF as an important tool to monitor response to end of antibiotic therapy as the same of spirometry. In particular, we demonstrate a significant correlation between the gold standard CF lung function parameter FEV1 and a small airways inhomogeneity index such as LCI 2.5.

Also Hatziagorou et al. investigated the use of LCI to assess IV antibiotic treatment response with a significant improvement in most lung function parameters: LCI (p = 0.0001), FEV1 (p = 0.05), FEV1 z-score (p = 0.033) and FEF25-75 (p = 0.046). LCI decreased by a mean of 1.77 lung turnovers with an average decrease in LCI of 26% (p = 0.001) and average increase in FEV1 of 10.36% (p = 0.05) post IV therapy for PE [28]. These data are more encouraging than ours but the caseload is much smaller (32 patients) and the evaluation was performed one month after IV antibiotic therapy [30]. We may hypothesize that a more distant evaluation time from initiation of antibiotic therapy may better stabilize lung function.

Our data are more in line with Robinson et al. that reported a LCI2.5 mean decrement of 0.48 lung turnovers with a 3.8% change and with Eef Vanderhelst that had described a significant decrease of 4.5% among only CF adult patients [22].

Our data suggest the hypothesis that this variability during PE is reduced for the presence of inflammation, mucus and increased broncostriction [31, 34].

Sacin and Scond are two values that are becoming more studied in literature, especially Sacin that reflects alveolar functionality, starting point of lung damage in CF disease. In contrast to Vanderhelst, our data demonstrate that LCI 2.5 correlates with both Sacin and Scond emphasizing how much in CF-PE both proximal and distal airways are affected [23].

**Fig. 2:**
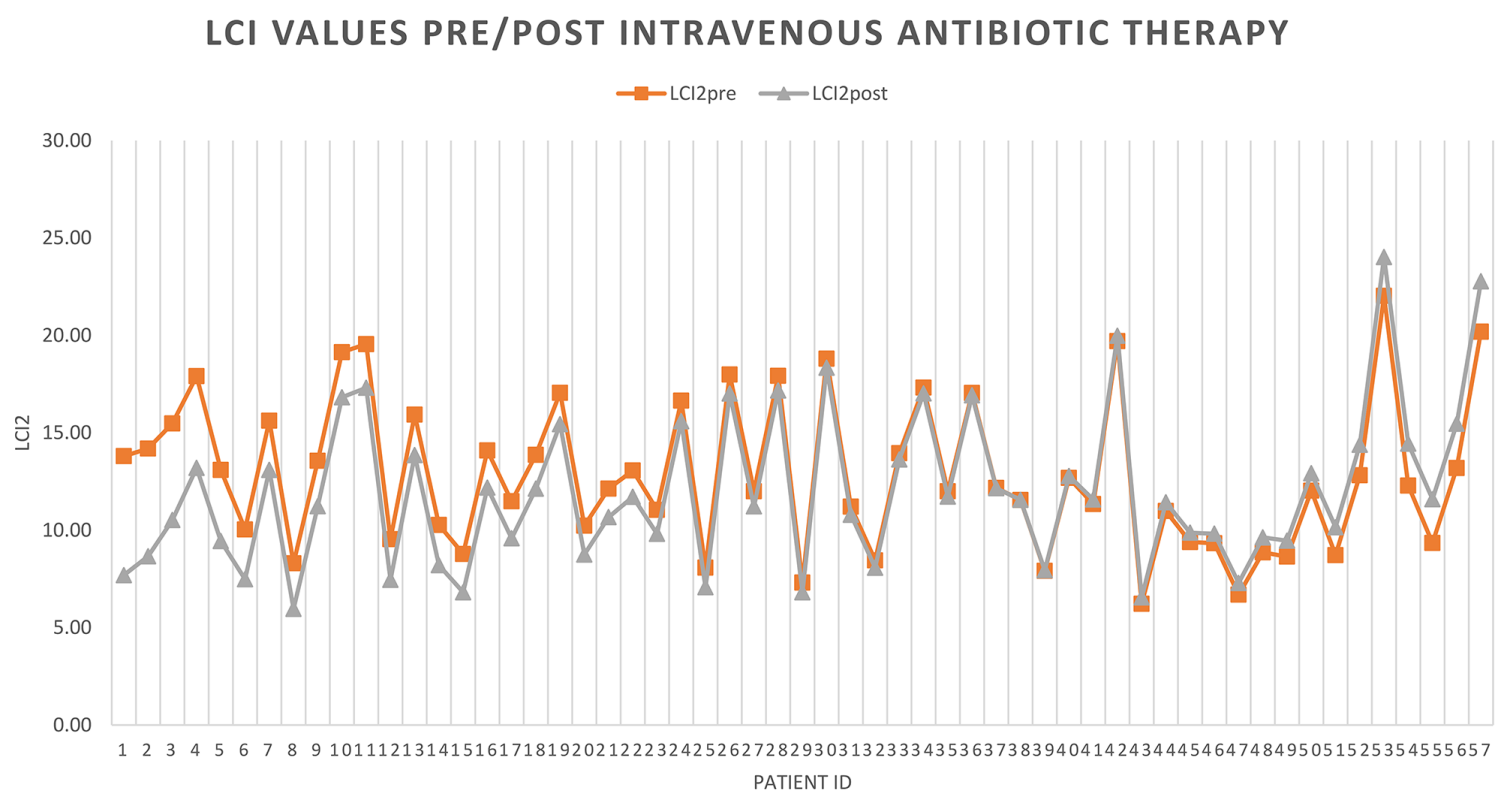
LCI values pre/post intravenous antibiotic therapy. This figure shows the LCI trend of 57 pwCF underlining the excellent results obtained after 14 days of intravenous therapy for pulmonary exacerbation

The first comment as literature reports it’s about the correlation between age and LCI values, infact these two variables increase proportionally as reported in Figs. 1 and 2 [11].

In our setting, LCI2.5 and FEV1 are concordant in showing a statistically significant amelioration of lung function after an IV antibiotic therapy. Despite its limits, we suggest that LCI 2.5 may be adopted as an additional biomarker to assess lung function response to antibiotic therapy, at the end of PE.

Focusing on the population with stability of LCI value we notice at first evaluation an higher average spirometric value than the general population. In our experience, one possible explanation might be that patients with better respiratory function experience fewer changes in ventilation homogeneity and probably less peripheral airway damage during exacerbation. Sub-population with improvement of LCI value post IV antibiotic therapy start the evaluation with an average of FEV1 lower between patients who are stable or worsened LCI value probably because the percentage of pseudomonas aeruginosa colonization is more represented and this aspect is line with what literature reported [35] underlining the correlation between pseudomonas aeruginosa with the decline of FEV1. About patients of sample size with worsening of LCI, according our experience it’s necessary an observational long time to establish the possible reason of these LCI results.

According our experience, we focused attention on 4 patients who showed a greater drop in LCI 2.5 (> 15%) and analyzing their clinical conditions we try to explain this anomalous trend. One of these patients had Allergic Bronchopulmonary Aspergillosis, spirometric indexes improved but LCI value did not respond; one pediatric patient did not respond probably for her severe anatomic compromission, with computed tomography confirming severe atelectasis in upper lobe of the right lung. Two adult women patients from starting to the end of hospitalization preserved more or less the same spirometric values, including FEF 25–75% for small airways, despite primary value of LCI 2.5 and secondary inhomogeneity parameters had a severe worsening.

About our experience and expertise on administration of MBW we tried to explain this no response; one of the possibilities was performing hypertonic solution or Dornase alfa shortly before the execution of test with an increased mobilization of mucus and poor compliance of some patients with daily respiratory physiotherapy.

A major limitation to our study is the lack of a baseline LCI 2.5 value in well-being due to SARS-CoV-2 pandemic with reduction of outpatient evalutations and of the routine availability of Exhalyzer–D in our center only after March, 2020. An other limitation of our study is to calculate LCI not in well-being but already in patients during pulmonary exacerbation. For each patient we don’t have a previous analysis in stability compared to the exam done at the admission for PE. Among limitations were the lack of knowledge about aerosol therapy treatment (Dornase alfa, hypertonic solution, bronchodilators) performed during the hospitalization for IV antibiotic treatment, and the typology of chest physiotherapy administered by respiratory therapists. Sovrainfections could represent another possible bias factor that prevented improvements of LCI 2.5 values.

The variability of LCI response for each patient (improvement vs. stability vs. worsening) could be explained by the presence of hypothetical different clusters of PE in CF, as recently reported in the literature for adult patients, but further studies are needed to prove this hypothesis [36].

## Conclusions

In conclusion, our study shows that LCI is a valid tool to monitor antibiotic responses in PE in CF in the same way as the FEV1, with the advantages of being easy to perform especially in pediatric population. We can assume that LCI will be used in conjunction with the clinic, to be evaluated at the end of antibiotic cycle to make any treatment implementations or total changes. So LCI can be a useful tool to use together with clinical practice at the end of antibiotic IV therapy to understand if the drugs clinical choise had been appropriated, but also if physicians have to switch and change antibiotic drugs, or if even they must extend the duration of antibiotic IV therapy for patients with CF.

Future long-term studies are needed to confirm these findings and to identify an agreed change to monitor response to therapy.

## Data Availability

The datasets used and/or analysed during the current study are available from the corresponding author on reasonable request.

## Abbreviations

MBW: Multiple Breath washout
CF: Cystic Fibrosis
LCI: Lung clearance index
FEV1: Forced expiratory volume in 1 s
CFTR: Cystic fibrosis transmembrane conductance regulator
PE: Pulmonary exacerbation
IV: Intravenous
FVC: Forced vital capacity
FEF25-75: Forced expiratory flow 25–75%
PPaa: Pediatric and Pre-adult age
